# Detection of Diagnostic Antibodies in Immune‐Mediated Diseases: A Focus on Antigens and Technologies

**DOI:** 10.1002/cbic.70354

**Published:** 2026-04-30

**Authors:** Silvia Bracci, Feliciana Real‐Fernandez, Federico Pratesi, Francesca Nuti, Paolo Rovero, Anna Maria Papini

**Affiliations:** ^1^ Interdepartmental Research Unit of Peptide and Protein Chemistry and Biology (PeptLab) and Center of Competences in Molecular Diagnostics for Life Sciences (MoD&LS) University of Florence Sesto Fiorentino Italy; ^2^ Department of Chemistry “Ugo Schiff” University of Florence Sesto Fiorentino Italy; ^3^ CNR ‐ Istituto di Chimica dei Composti Organometallici (CNR‐ICCOM) Sesto Fiorentino Italy; ^4^ Department of Translational Research and of New Surgical and Medical Technologies University of Pisa Pisa Italy; ^5^ Department of Neurosciences, Psychology, Drug Research and Child Health Section of Pharmaceutical Sciences and Nutraceutics University of Florence Sesto Fiorentino Italy

**Keywords:** autoantibodies, autoimmune diseases, immunoassays, peptide and protein antigens, serological biomarkers

## Abstract

Autoimmune diseases are conditions characterized by aberrant B‐cell and T‐cell reactivity against self‐antigens. Autoantibodies are serological biomarkers of autoimmune diseases, as such, autoantibody testing is a key step for diagnosing and classifying many autoimmune diseases, as well as monitoring disease activity and devising a treatment strategy. Considering the rising number of people affected by autoimmune diseases worldwide, it is even more important to have efficient techniques that combine high sensitivity and specificity with reduced sample processing times and an automated high‐throughput workflow. In this context, the identification and validation of new autoantigens and autoantibodies, together with the implementation of technological advancements, has led, in the last decades, to an improvement in patient diagnosis and stratification. Here, we review the major antigens of some of the most common autoimmune diseases, and the most widely used assays employed in diagnostic laboratories for the detection of their cognate antibody, confronting more traditional platforms with emerging ones in selected cases of study.

## Graphical abstract

This review explores autoantibody detection in immune‐mediated diseases, highlighting autoantigens including post‐translational modifications. We compared enzyme‐linked immunosorbent assay and indirect immunofluorescence with automated chemiluminescence platforms. These innovations refine diagnostic accuracy and patient stratification, supporting personalized medicine.

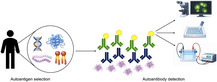

## 1 Introduction

The population affected by immune‐mediated diseases (IMDs) is estimated to be around 10%, and epidemiological data suggest evidence of a steady rise in their incidence and prevalence over the last decades, which are expected to keep increasing worldwide [1, 2, 3, 4].

Despite notable progress in diagnosing and treating IMDs, the health and economic burden imposed by these conditions remains substantial [4, 5, 6]. Effective and reliable diagnostic and prognostic assays are essential for an early diagnosis and monitoring of disease activity to reduce morbidity, disability, mortality, and improve quality of life [7, 8, 9].

Autoimmune diseases are a diverse group of conditions characterized by the presence of autoreactive B and/or T cells that evade tolerance and are activated against the body itself, causing organ‐specific (type 1 diabetes, autoimmune liver diseases, celiac disease (CD), etc.) or systemic (systemic lupus erythematosus (SLE), rheumatoid arthritis (RA), etc.) chronic inflammation and tissue damage [10, 11, 12, 13, 14]. Although both B‐ and T‐cells contribute to the loss of tolerance, identifying T‐cell antigens is more challenging, and T‐cell‐based assays are not routinely performed, given the greater technical challenge of using live cells. Thus, a critical role in the diagnosis of autoimmune diseases is played by disease‐specific autoantibodies, that can be detected through many validated assays in serum samples, which have the advantage of being obtained through minimally invasive blood draws [13, 15, 16]. Autoantibodies also present the advantage of being disease biomarkers for autoimmune conditions: some are predictive of the development of the disease (as antimitochondrial antibodies (AMA) for primary biliary cholangitis (PBC) and anticitrullinated peptide antibodies (ACPA) for RA), while others fluctuate according to disease activity and thus have a prognostic value (as antiglomerular basement membrane antibodies in Goodpasture's syndrome) [17, 18, 19, 20]. Nonetheless, even if the measurement of autoantibodies is a major diagnostic tool in autoimmune diseases, autoantibodies can often also be found in healthy individuals. So, it is important that autoantibody testing is performed for patients who have a reasonable likelihood of having the disease [21, 22].

Protein post‐translational modifications (PTMs), such as phosphorylation, glycosylation, citrullination, and ubiquitination, are essential for increasing proteome diversity and regulating protein activity, structure, localization, and interactions, thus influencing numerous vital biological processes [23]. However, dysregulation of the spontaneous or enzymatic processes controlling PTMs, often driven by inflammatory conditions, can result in aberrant or excessive modifications generating neoantigens, that can lead to a breach of tolerance, with induction of antibodies or autoreactive T cells, and the final consequence of an autoimmune response [13, 24, 25, 26]. While genetic background creates a predisposition to autoimmune diseases, environmental factors, such as exposure to infectious microorganisms or chemicals, appear to be necessary for initiating or exacerbating autoimmune response and clinical disease manifestation [11, 13, 27, 28, 29, 30]. In this context, recombinant or extracted protein antigens may only partially reproduce the correct modification essential for antibody recognition. On the other hand, synthetic peptides can be produced in high quality and quantity through standard and reproducible synthetic protocols, enabling the straightforward introduction of nonstandard, modified amino acids corresponding to the target PTM into the sequence to mimic the native epitope [31, 32, 33, 34].

The history of autoantibodies began over a century ago with the introduction of Ehrlich's concept of “horror autotoxicus” and the development of the Wassermann test for syphilis. Still, progress in identifying and detecting autoantibodies slowed until the 1950s, when indirect immunofluorescence (IIF) was first described by Coons, Kaplan, and Weller. This technique is still considered the “gold standard” for screening antinuclear antibodies (ANAs). Remarkable advances in identifying and cataloging the molecular targets of autoantibodies led to a new generation of platforms such as enzyme‐linked immunosorbent assay (ELISA) and western blot (WB), followed more recently by multiplexed analyte immunoassays like addressable laser bead immunoassays (ALBIA), and chemiluminescent immunoassays (CLIA) that potentially allow for the simultaneous detection of multiple autoantibodies, providing a comprehensive overview of the patient's autoimmune profile in a single test [35, 36, 37, 38, 39]. Biosensor technologies are rapidly emerging as less complex, faster, and lower‐cost alternatives to traditional methods that require skilled personnel and well‐equipped laboratories. Furthermore, biosensors, with their high sensitivity, specificity, and possible high throughput and point‐of‐care applications, could, in the future, revolutionize the field of IMD diagnosis. However, while these newer technologies have high potential, they need to be perfected and thoroughly evaluated before widespread clinical adoption [40, 41, 42].

Here, we address the antibody identification in autoimmune diseases outlining both methodological considerations and the fundamental influence of antigenic structure, localization, and biochemistry on antibody recognition across several autoimmune diseases. This article reviews some of the most widely used technologies used in clinical practice, taking examples from specific autoimmune diseases or antibodies for which specific antigens are particularly relevant and certain techniques which are gold standard or common practice, with a focus on comparing the performance of assays based on different antigens that use more traditional or newer technologies. We acknowledge that, due to the vast amount of available literature in this field, which is ever evolving, this review is not exhaustive.

## 2 Types of Immunoassays

### 2.1 Immunofluorescence on Tissues or Cells

Immunofluorescence uses fluorescent‐labeled antibodies to detect and visualize specific antigens within biological samples, such as cells or tissue sections, preserving their spatial organization, making the technique particularly valuable for detecting autoantibodies directed against intracellular or membrane‐associated targets and for identifying characteristic staining patterns associated with specific autoimmune diseases.

After appropriate preparation, the sample is incubated with a primary antibody that specifically binds to the target antigen.

In direct immunofluorescence (DIF), the fluorescent dye is directly conjugated with the primary antibody. Instead, IIF employs an unlabeled primary antibody and a fluorescently labeled secondary antibody that binds to the primary antibody [43, 44]. While DIF is a quicker and more straightforward method, it can be less sensitive due to lower staining intensity if the antigen of interest is minimally expressed. On the other hand, the IIF approach allows for signal amplification and enhanced sensitivity, as multiple secondary antibodies can bind to a single primary antibody. After incubation and washing to remove unbound antibodies, the sample is examined using a fluorescence microscope, where the emitted fluorescence at specific wavelengths makes the target antigen visible [45, 46].

In autoimmune disease diagnosis, the aim is to obtain recognition between antigens and autoantibodies. Thus, direct IF requires a tissue sample from the patient to detect pathogenic autoantibody‐antigen complexes, and it is especially useful when the goal is to visualize immune complexes or antigen distribution within the native tissue, such as in autoimmune skin conditions. IIF is less invasive, detecting circulating autoantibodies (that act as the primary antibody) in biological fluids such as serum. IIF is widely used with substrates such as HEp‐2 cells for detecting autoantibodies against intracellular antigens, because these antigens are presented in their native cellular context with preserved conformation, PTMs, and molecular interactions. The resulting fluorescence patterns provide diagnostic clues that reflect the underlying antigen specificity. Only a limited number of intracellular autoantigens can be reliably produced in a recombinant form that preserves key epitopes, meaning solid‐phase assays cover only common targets, while rarer, structurally complex, or PTM‐dependent antigens remain detectable only through characteristic HEp‐2 IIF patterns [47, 48].

In cell‐based assays (CBAs) cultured cells, typically human embryonic kidney 293 (HEK293), are transfected with the human complementary DNA (cDNA) encoding the target antigen to be expressed on their surface. These assays are especially important for detecting autoantibodies directed against conformational epitopes of membrane proteins, which are often not preserved in denatured proteins used in conventional solid‐phase assays. Because the antigen is expressed in the cellular environment, CBAs preserve the native three‐dimensional structure and multimerization. This makes CBAs particularly suitable for detecting pathogenic autoantibodies targeting cell‐surface antigens, like those involved in autoimmune neurological conditions [49, 50, 51].

Patient serum, plasma, or cerebrospinal fluid (CSF) are then incubated with these cells, and antigen‐specific antibodies are identified by fluorescent secondary antibodies, that allow the determination of antibody levels by endpoint titration with fluorescence microscopy or quantitatively by flow cytometry [52].

Live cells present the correctly folded antigen, but the maintenance of living cells is technically demanding, and thus live CBAs are time‐consuming, and their use is limited to specialized centers. Commercial CBAs used by clinical laboratories, however, use fixed and permeabilized cells. Fixation with paraformaldehyde is performed to preserve and stabilize cells by protein cross‐linking and to allow transportation of the kits, but it may decrease antigenicity by masking or denaturing some of the epitopes; moreover, permeabilization of cells could result in false positive outcomes due to the identification of antibodies targeting intracellular epitopes. Thus, cell fixation and permeabilization could lead to nonspecific antibody binding, compromising assay sensitivity and specificity compared to live CBAs [49, 52, 53]**.**

### 2.2 Immunoblotting

Immunoblotting, also known as Western blotting, is a widely utilized technique in immunology and molecular biology for detecting specific proteins within a complex mixture, employing the specificity of the antibody–antigen complex. This method combines the principles of gel electrophoresis and antibody‐based detection of immunoassays to assist in the diagnosis and research of autoimmune diseases, as it enables the identification of autoantibodies against specific antigens [54].

The process of immunoblotting involves several key steps. First, the sample containing the target protein antigens undergoes electrophoresis, in which proteins are separated based on their molecular weight (using sodium dodecyl sulfate‐polyacrylamide gel electrophoresis (SDS‐PAGE)).

After electrophoresis, the proteins are transferred from the gel onto a nitrocellulose or polyvinylidene fluoride (PVDF) membrane, which is the blotting step. Once transferred, the membrane is incubated with the biological sample potentially containing a primary antibody specific to the target protein, generally a serum or plasma sample, that binds to its corresponding antigen on the membrane. After washing away unbound primary antibodies, an enzyme‐labeled secondary antibody conjugate is applied to reveal the antibody–antigen complex, forming the protein band [55].

Similar techniques to immunoblotting include dot blotting and line blot. Dot blotting is a simplified version of immunoblotting where proteins are directly applied onto a membrane in a dot pattern. In line blots, highly purified recombinant or native antigens are fixed onto nitrocellulose or PVDF membrane strips in thin parallel lines. These techniques are less time‐consuming as they do not require electrophoresis, making them useful for the rapid screening of multiple samples [56, 57], but are based on the use of recombinant proteins, whereas western blotting onto SDS‐PAGE separated samples allows the detection of antibodies directed against antigens in their native form. Anyway, DOT blot and LIA give higher sensitivity and simplicity and allow faster multiparametric analysis of several antigens. Commercial kits are available for the detection of many different antibody specificities related to various autoimmune diseases [58].

However, immunoblotting techniques offer only qualitative, or possibly semiquantitative, results, providing a relative comparison of protein levels and not an absolute measure of concentration [55, 56]. Qualitative results can be obtained by simply noting the presence or absence of a spot by visual inspection. Scanning densitometry is considered the golden standard for the semiquantitative evaluation of blots, obtained by analyzing the intensity of the bands and yielding a result relative to the intensity of respective positive and cutoff controls [57, 59].

### 2.3 Solid‐Phase Platforms

Initially developed to replace radioimmunoassays, solid‐phase assays offer a safer, more accessible alternative by eliminating the need for radioactive labeling of antigens or antibodies and thus the special equipment required, but without losing the high sensitivity of RIAs [60]. This, and the possibility of standardization and automation of these techniques, has led to their widespread use in clinical laboratories worldwide [61]. In these assays, antigens are immobilized on a solid surface, which may partially alter their native structure; therefore, solid‐phase assays are particularly effective for detecting autoantibodies directed against linear or structurally stable epitopes that remain accessible after antigen purification and immobilization, whereas antibodies recognizing conformational or complex epitopes may be less efficiently detected.

The ELISA was introduced in the 1970s [62] shortly after techniques for labeling antibodies with enzymes were reported, while in the last decades, advancements in the automation of diagnostic platforms have led to the swift implementation of solid‐phase assays such as CLIA and fluorescent enzyme immunoassays (FEIA) in clinical laboratories. These new technologies offer significant improvements in sensitivity and specificity, as well as higher throughput and shorter turnaround times compared to more traditional methods.

Multiplex autoantibody assays enable the simultaneous detection of multiple autoantibodies. This introduces the possibility of performing comprehensive autoantibody profiles, which can be particularly useful in improving the diagnosis of autoimmune diseases, helping in the patients’ stratification, especially in the case of diseases in which multiple antibodies are involved. Antibody profiling can contribute to an earlier diagnosis, due to the detection of predictive antibodies, and also to the classification of patients into the correct disease subtype, characterized by specific autoantibodies. They can also assist with monitoring of disease activity and outcomes, as some antibodies are linked to certain disease prognosis and thus in treatment selection. Moreover, multiplex assays, when used in follow‐up testing, allow the detection of several clinically relevant antibodies that are useful in differential diagnosis and prognosis but are not routinely analyzed [63, 64]. Theoretically, since the principle is the same, results for antibody specificities obtained with multiplexed assays should be comparable to those obtained with conventional techniques. However, combining multiple antigens within a single assay may result in suboptimal conditions for individual antigen–antibody interactions and can introduce interference that affects signal detection [65, 66].

The main concern surrounding multiplex immunoassays for clinical and research use relates to their analytical quality and validation. Many multiplex assays are introduced to the market with limited validation against conventional or gold‐standard methods, highlighting the ongoing need for rigorous performance evaluation before widespread clinical adoption.

Compared with singleplex assays, multiplex platforms introduce substantially greater quality control challenges, partly because quality control materials and standardized algorithms for interpreting multiplex data are still insufficiently developed. The simultaneous measurement of multiple heterogeneous analytes requires the generation of robust and meaningful calibration curves under a shared set of assay conditions, yet identifying a single sample dilution factor that accommodates the physiological range of all markers is often impractical. This frequently necessitates splitting panels according to analyte abundance, complicating workflow and limiting standardization. Moreover, optimizing a common assay format, including dilution factors, capture and detection systems, incubation times, and washing conditions, that performs adequately for every constituent marker, represents a major obstacle to harmonization and automation. As a result, the likelihood that all analytes within a multiplex panel simultaneously meet quality control specifications is considerably lower than for a singleplex assay. Additional challenges arise from antibody validation: large‐scale characterization of affinity, specificity, cross‐reactivity, and kinetic parameters is labor‐ and cost‐intensive, and antibodies validated in singleplex formats may exhibit cross‐reactivity when used in multiplex settings, necessitating application‐specific validation [67]. Cross‐reactivity can reduce the limit of detection and increase the risk of inaccurate or misleading results. Furthermore, studies have shown that as the number of analytes in a panel increases, nonspecific background and interanalyte interference may compromise analytical sensitivity and specificity compared to corresponding singleplex assays, further emphasizing the complexity of ensuring reliable performance in multiplex immunoassays.

Cross‐reactivity represents a major limitation of multiplex immunoassays, as antibodies frequently bind unintended targets due to imperfect specificity, generating signals that are indistinguishable from true antigen‐antibody. Therefore, in the most favorable scenario, cross‐reactivity contributes primarily to increased background noise, which compromises analytical sensitivity, whereas in the worst case, it produces false‐positive signals that may lead to erroneous conclusions and misclassification. This problem becomes progressively more severe as the number of analytes in a multiplex panel increases, because the number of potential cross‐reactive interactions rises approximately with the square of the number of targets (4N^2^), dramatically expanding the number of unintended antibody–antigen, antibody–antibody, and reagent interactions that can occur. As a result, even a single poorly specific antibody or contaminating reagent can compromise the performance of the entire panel, highlighting cross‐reactivity as a fundamental barrier to scaling multiplex assays and a key factor limiting their sensitivity, specificity, reproducibility, and overall analytical reliability [68, 69]*.*

Many clinicians may wrongly assume that commercially available immunoassays, including multiplex platforms, are fully validated and equivalent to conventional methods, underscoring the importance of understanding each assay's limitations within the specific population being tested [65].

One of the most common multiplex techniques is ALBIAs, while many others, such as particle‐­based multianalyte technology (PMAT), nanobarcodes, antigen arrays, and biosensors are emerging but are not widely adopted [19, 35, 63]. These new techniques are becoming increasingly important in the field of autoimmune disease diagnostics and are slowly substituting more traditional solid‐phase assays in the detection of many autoantibodies [35, 70, 71].

#### 2.3.1 Enzyme‐Linked Immunosorbent Assay (ELISA)

ELISA works by employing enzymes as labels to detect the presence of a specific analyte in a sample: upon adding the appropriate enzyme substrate, a colorimetric reaction occurs which is monitored through a spectrophotometric analysis to quantify the analyte. The basic procedure involves the initial coating of the antigen or antibody in the plastic surface (now microtiter plate wells), adding the sample, and then introducing an enzyme‐labeled antibody specific to the analyte in question [72].

Different ELISA protocols can be used in clinical practice depending on the nature of the sample and analyte [73].

Direct ELISA involves the adsorption of the antigen on the solid support, followed by the enzyme‐labeled primary antibody. Then, the substrate is added to the well, and the final readout signal can be recorded after stopping the reaction. A major limitation of this approach is the availability of the enzyme‐labeled antibody, a problem which is solved using indirect ELISA: the antibody conjugated with the enzyme is a secondary antibody that recognizes the primary one, and not directly the antigen coated on the wells. In the case of an autoantibody assay, the primary antibody is in the analyte to be detected in the serum/plasma of patients. This method determines (or allows) the amplification of the signal because multiple secondary antibodies can bind to the primary antibody, increasing sensitivity. Sandwich ELISA involves the initial coating of an antibody (capture antibody) onto the well bottom before incubating the sample containing the analyte. Then, another analyte‐specific antibody (detection antibody) labeled with the enzyme, is added and is followed by the substrate to reveal the concentration of the target molecule. Because of the double recognition and the general use of a monoclonal antibody as capture and of a polyclonal as detection, sandwich ELISA usually presents a high sensitivity and specificity, and it is useful to detect analytes present in very low concentrations. In competitive ELISA, the surface of the wells is coated with the antigen or antibody. The sample containing the analyte and the enzyme‐linked counterpart to the adsorbed molecule are incubated together and then placed into the well simultaneously. The labeled and unlabeled antigen or antibody molecules compete to bind to the antibody in the sample. In this case, there is an inverse proportion between the analyte concentration and the intensity of the resulting coloration.

Several factors influence the sensitivity of an ELISA, the first being the affinity of the antibodies for their target antigens as high‐affinity antibodies enable the detection of lower antigen concentration. Different ELISA protocols can also affect sensitivity. Signal amplification given by enzyme‐linked secondary antibodies can further enhance the assay sensitivity. Sandwich ELISA generally determines higher sensitivity than other protocols due to the dual recognition between the antigen, the monoclonal capture antibody, and the polyclonal detection antibody. The choice of enzyme‐substrate system is another critical factor: reactions that produce an intense and easily measurable signal improve the assay sensitivity, allowing for the detection of lower quantities of analyte [74]. The most used enzymes are horseradish peroxidase (HPR), alkaline phosphatase (AP), β‐galactosidase, and glucose oxidase [72].

The specificity of an ELISA depends mainly on the quality of the antibodies, the use of appropriate blocking agents to prevent nonspecific binding, thorough washing steps to remove unbound substances, and the selection of enzyme‐substrate systems that produce minimal background noise [74].

#### 2.3.2 Chemiluminescent Immunoassay (CLIA)

Luminescence consists in the emission of light generated when a molecule in an excited state relaxes to its ground state. In chemiluminescence, the energy to excite the molecule is produced by a chemical reaction [75]. CLIA uses a luminescent molecule as the label to indicate the presence of the analyte. The antigens are bound to the solid phase, usually paramagnetic beads, to obtain a larger surface binding area and to avoid the use of microtiter plates [76]. In direct CLIA, the luminophore, usually acridinium and ruthenium esters, is conjugated to the secondary antibodies, while in indirect CLIA, substrates such as 1, 2‐dioxetane aryl phosphate (AMPPD) or isoluminol are turned into luminescent molecules by reaction catalyzed by enzymes (AP or HRP, respectively) conjugated to secondary antibodies. Thus, the luminescence signal is proportional to the antigen‐specific antibody titer. CLIA has many advantages: low background or interfering emission allows for high sensitivity and specificity, and a wide dynamic range; high automation and standardization determine increased productivity and reproducibility [77].

#### 2.3.3 Fluorescent Enzyme Immunoassay (FEIA)

FEIA is an enzyme immunoassay in which the secondary antibody is conjugated to an enzyme that transforms the substrate into a fluorescent molecule, which allows for more rapid measurement of small concentrations of antigens compared to ELISA [78]. The substrate is usually 4‐methylumbelliferyl‐β‐D‐galactoside, and antibodies are labeled with β‐galactosidase [79, 80]. As with CLIA, FEIA allows for a high degree of automation and thus shorter turnaround times and increased productivity of the diagnostic laboratory [77].

#### 2.3.4 Multiplex Addressable Laser Bead Immunoassays (ALBIA)

ALBIAs are based on the Luminex technology [81]. This technique employs sets of beads characterized by 100 unique colors resulting from the combination of two fluorescent dyes at specific ratios; to allow multiplexing, different antigens are bound to beads of different colors. The beads are then combined in microtiter plate wells to allow incubation of the different antigens with patient sera and then with a fluorochrome‐labeled secondary antibody. The beads are analyzed through flow cytometry with a double laser system. A red laser excites the dyes in each bead to identify the dye ratio. The green laser excites the fluorescent label to quantify the captured analyte [82, 83]. ALBIA is a fast, cost‐effective, quantitative, and reliable method for detecting multiple autoantibodies against different target autoantigens simultaneously and can be a valid alternative to other solid‐phase assays such as WBs or ELISA [81]. However, the technique requires a flow cytometer, an instrument not always available.

### 2.4 Surface Plasmon Resonance (SPR)

Surface plasmon resonance (SPR)‐based immunoassays record changes in the refractive index or thickness of the sensor surface with the excitation of light to detect the biorecognition analyte, in real‐time and without the need for secondary labels [84]. Antigens are immobilized on a gold sensor chip surface, whereas the analytes flow in solution. The full automatization and the fast response are some of the major points of SPR, together with the possibility to detect low‐affinity antibodies that can be underestimated in other techniques like ELISA, as described for antiglucosylated peptide antibodies in MS [85].

Autoantibody detection by SPR‐biosensors has been described in the case of antiphospholipid syndrome (APS) [86], RA [87], SLE [88, 89], multiple sclerosis (MS) [90], and CD [91]. A remarkable example in which SPR has been applied in AD is the study of Trabucchi et al. to characterize insulin autoantibodies for type 1 diabetes in terms of concentration and affinity showing different pattern of proinsulin autoantibodies [92]. In fact, comparative tests between childhood‐onset and adult‐onset diabetic patients indicated a distinct autoimmune process among diabetic patients.

SPR is used as a powerful technique for a deep understanding of the kinetic parameters of antigen–antibody interaction that can help in patient stratification, although up to now it has not definitively entered routine marker screening.

## 3 Detection of Autoantibodies in Autoimmune Disorders

### 3.1 Autoimmune Bullous Skin Diseases and Their Antigen Specificities

Autoimmune bullous diseases (AIBDs), characterized by blistering of the skin and mucous membranes, are caused by autoantibodies targeting keratinocyte adhesion molecules. In the case of pemphigus diseases, the major target antigens are cadherin‐type desmosomal proteins, specifically desmogleins (Dsg1 and/or 3, depending on the disease subtype), which connect adjacent keratinocytes [93, 94].

**FIGURE 1:**
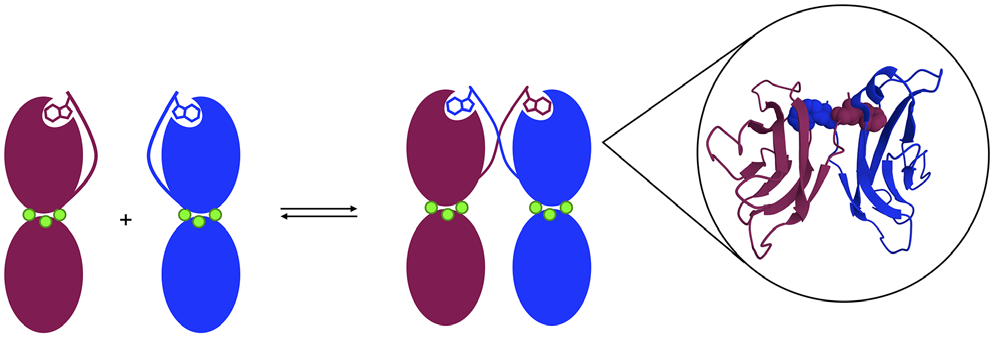
Schematic representation of the cadherin strand‐swapping process. Two monomers (burgundy and blue) are schematically shown on the left in a closed conformation and as the strand‐swapped dimer on the right. Only the EC1 and EC2 domains are represented, with calcium ions (green) in between. (PDB: 2O72).

Cadherins form a large superfamily of cell‐surface receptors that mediate calcium‐dependent cell–cell recognition and adhesion. All members share a conserved structural unit, the EC domain, an approximately 110‐residue β‐fold domain, and are classified into subfamilies based on the number and arrangement of these repeats. Linkers between successive EC domains bind three Ca^2+^ ions, which stabilize the structure and confer the characteristic curvature to the ectodomain. At adherens junctions, cadherins cluster and establish adhesion through a combination of *cis* (same‐cell) and *trans* (intercellular) interfaces, with the latter formed by ectodomains that extend across the intermembrane space. Classical cadherins engage in trans adhesion by forming strand‐swapped dimers through their membrane‐distal EC1 domains: the N‐terminal A* strand of one molecule inserts into a conserved hydrophobic pocket of its partner, stabilized by an anchor tryptophan residue (Figure 1). This interaction exemplifies 3D domain swapping, where the A* strand can dock intramolecularly to form a closed monomer or intermolecularly to generate a swapped dimer [95]. Blister formation in pemphigus is thought to occur through two main mechanisms: steric hindrance, where pathogenic autoantibodies directly block trans or cis interactions of desmogleins, or through cellular responses, in which autoantibodies trigger internalization and degradation of desmogleins.

Pathogenic autoantibodies mainly recognize the N‐terminal EC domains of these transmembrane proteins, which are essential for Dsg interaction [96, 97, 98, 99].

In the case of pemphigoid diseases, the antigenic targets are hemidesmosomal proteins and structural filaments, resulting in the separation of the epidermis from the underlying dermis [48, 100, 101]. In bullous pemphigoid (BP), autoantibodies are directed toward collagen XVII (BP180) and dystonin‐e (BP230) [102, 103]. BP180 is a transmembrane glycoprotein with a C‐terminal extracellular portion composed of 15 collagen domains, each separated by noncollagenous (NC) domains. The collagen domains consist of a repeating glycine‐X‐Y motif, where X is proline and Y is hydroxyproline. The formation of a collagen triple helix makes BP180 a trimer. Besides several potential intracellular sites, BP180 also contains a rare phosphorylation site at an extracellular serine residue. Specifically, serine 544 within the human BP180 16th NC domain, NC16, is phosphorylated by casein kinase II, which enhances its recognition as an epitope for BP autoantibodies and may play a regulatory physiological role [104, 105]. Indeed, the NC16 is the key antigenic site targeted by IgG autoantibodies in most patients. As for BP230, an intracellular protein of the plakin protein family, the major epitope is located in the globular C‐terminal domain [106, 107]. Anti‐BP230 autoantibodies are believed to arise through an epitope spreading mechanism: antibodies against extracellular epitopes of BP180 can cause cellular damage, exposing intracellular BP230 and thereby promoting the development of anti‐BP230 autoantibodies during the disease course. It is possible that the N‐terminal region of BP230, essential for its localization to the hemidesmosome via interactions with BP180 and the β4 integrin subunit, may be the first antigenic site recognized, followed later by the C‐terminal through epitope spreading [108, 109].

#### 3.1.1 Performance of Immunofluorescence Techniques in the Case of AIBDs

DIF performed on cryosections of perilesional biopsy specimens is considered the gold standard for diagnosis, with a specificity of 98% and sensitivities ranging from 82%–91% [48, 110, 111, 112]. The fluorescence pattern observed is indicative of the disease subclass: intercellular deposits of IgG and/or C3 that show a honeycomb‐like pattern are characteristic of pemphigus foliaceus (PF), pemphigus vulgaris (PV), and other pemphigus type diseases, while BP and other rare subtypes present linear IgG and/or C3 deposits along the basement membrane zone (BMZ) [113, 114].

IIF detects circulating autoantibodies in the patient's serum on specific substrates, such as human skin, monkey esophagus, and rodent or monkey bladder. With pemphigus serum, monkey esophagus yields a sensitivity of 81%–100% and a specificity of 89%–100%, making it the optimal substrate in the screening for intercellular antibodies in suspected cases of PV and PF [48, 115, 116]. Salt‐split skin is particularly useful for the detection of anti‐BMZ autoantibodies, with a sensitivity of 73%–96% and a specificity of 97%. It is also helpful in determining the type of pemphigoid disease based on the location of the antigens on the top or bottom of the split [48, 112, 117]. The serological diagnosis of AIBDs is a multistep process that begins with IIF as the gold‐standard screening to observe fluorescence patterns, but requires follow‐up with antigen‐specific immunoassays, such as widely available ELISAs or specialized immunoblotting and immunoprecipitation, to identify the specific autoantibody specificities needed to confirm the diagnosis [110].

#### 3.1.2 Perspectives in IIF for AIBDs

The substrates commonly used for IIF comprise a wide variety of antigens. To simplify interpretation, recombinant IIF assays have been developed in which only one antigen is evaluated. The substrates, either HEK293 cells that recombinantly express the selected antigen or the purified antigen itself, are coated onto miniature BIOCHIPs (Euroimmun, Lübeck, Germany) which are arranged on microscope slides. Different substrates can be combined in a mosaic BIOCHIP to screen multiple antigens in a single incubation, saving time and serum, and allowing differentiation among the various disease subtypes, all using a single sample. This BIOCHIP technology was found to afford results comparable to the standard and time‐consuming multistep approach and thus is a practical, specific, and sensitive diagnostic alternative for AIBD screening with IIF and ELISA [115, 118, 119, 120]. Although ELISA and IIFT‐BIOCHIP show comparable diagnostic accuracy for pemphigus and BP, IIFT‐BIOCHIP is only semiquantitative, cannot reliably monitor antibody levels, and relies on subjective interpretation, which limits its clinical utility. ELISA, by contrast, offers high specificity, sensitivity, quantitative detection, and suitability for high‐throughput testing, but it is sensitive to operational conditions, prone to cross‐reactivity, restricted in detection range, costly, dependent on strict sample quality, and unable to directly detect antigen–antibody complexes.

#### 3.1.3 ELISA in AIBDs

ELISAs for the detection of antibodies to the main antigens in AIBDs are widely available, including anti‐Dsg1 and Dsg3 ELISAs, which have high sensitivity and specificity for pemphigus‐type diseases and help discriminate between them [121, 122, 123]. For BP, ELISAs for the detection of antigens BP180 and BP230 are available: BP180 ELISA has a much higher diagnostic utility compared to BP230 ELISA, reflecting the fact that BP230 autoantibodies are present in only around 60% of all BP patients. It is important to note that the combination of the two assays slightly increases the sensitivity of BP diagnosis over BP180 ELISA alone [48, 124, 125]. Moreover, ELISAs are not only useful for diagnosis but also for monitoring the disease course, as antibody levels often correlate with disease activity [125, 126, 127, 128, 129].

To further improve and standardize the serologic diagnosis of AIBDs, multivariant ELISA were developed using 6 major recombinant target antigens of pemphigus (desmoglein 1, desmoglein 3, envoplakin) and pemphigoid diseases (BP180, BP230, type VII collagen), plated in different wells. Two kits are now commercially available (MBL, Euroimmun), which allow the simultaneous screening of the different AIBD‐relevant antigens. Their multivariant nature, combined with the ELISA platform, enables a more objective, fast, and standardized screening compared to IF techniques. Their performance is comparable to that of the individual ELISAs in terms of sensitivity and specificity, and thus, they offer an alternative screening tool to the gold standard techniques and the multistep testing strategy. However, sharing the same cutoff values for all antigens may yield some unclear results compared to monovalent ELISA [122, 130, 131].

In the context of antigen‐specific assays, CLIA addresses some of the limitations of ELISAs by providing higher sensitivity, a broader dynamic range, and precise antibody quantification for monitoring disease activity. In addition, its streamlined and automatable workflow improves efficiency, while cost‐effective equipment and reagent use make CLIA particularly advantageous for large‐scale clinical testing [132]**.**

### 3.2 Antinuclear Antibodies (ANA) in Systemic Rheumatic Diseases

ANA are a diverse group of autoantibodies that target cellular components such as nucleic acids (DNA or RNA), proteins and their complexes. Certain ANAs are strongly related to specific diseases, known as ANA­associated rheumatic disease (AARD), such as SLE, systemic sclerosis (SSc), Sjögren syndrome (SjS), and idiopathic inflammatory myopathies (IIMs) among others (Table 1) [11, 71, 133].

**TABLE 1:** Autoantigen specificities and HEp‐2 IIF patterns of ANA associated with various rheumatic diseases.

Associated disease	Antibody specificity	HEp‐2 IIF staining
Systemic lupus erythematosus	Double‐stranded DNA	Nuclear homogeneous
	Nucleosome or chromatin	
	Smith (Sm) antigen (SmB/B’/N, SmD1–3, SmE, SmF, SmG)	Nuclear large/coarse speckled
	Ribosomal P (P0, P1 and P2 phosphorylated proteins)	Cytoplasmic dense fine speckled (can be missed)
	Proliferating Cell Nuclear Antigen (PCNA)	PCNA‐like (pleomorphic speckled)
Systemic sclerosis	Topoisomerase I (Scl70)	Topo I‐like
	Centromere protein B (CENPB)	Centromere
	RNA polymerase III	Nuclear speckled
	Fibrillarin	Clumpy nucleolar; perichromosomal staining in mitotic HEp‐2 cells
	Human RNase mitochondrial RNA processing (MRP) complex (Th/To)	Homogeneous nucleolar
	Nucleolar Organizer Region 90 (Nor90)	Punctate nucleolar; staining in the nucleolar organizer regions during metaphase
	U11/U12 ribonucleoproteins (U11/U12 RNP)	Nuclear large/coarse speckled
Sjögren syndrome (SjS)	SSB/La protein	Nuclear fine speckled
No specific disease	ssDNA	Nuclear homogeneous
Drug‐induced lupus, SLE, JIA and various other diseases	Histone	Nuclear homogeneous
SjS and SLE	SSA/Ro60 protein	Nuclear speckled
Idiopathic inflammatory myopathies	Histidyl‐tRNA synthetase (Jo1)	Cytoplasmic fine speckled
	Synthetase PL7, PL12, OJ, EJ, KS, Ha or Zo	
	Signal recognition particle (SRP)	
	3‐hydroxy‐3‐methylglutaryl‐coenzyme A reductase (HMGCR)	Cytoplasmic fine speckled (in a subset of cells)
	Antimelanoma differentiation‐associated protein 5 (MDA5)	
	Transcriptional intermediary factor 1 γ (TIF1γ)	Nuclear fine speckled
	Small ubiquitin‐like modifier activating enzyme (SAE)	
	Nucleosome‐remodeling deacetylase (NuRD) complex proteins (Mi2)	
	Antinuclear matrix protein 2 (NXP2)	Nuclear multiple dots

IIF for ANA testing is performed on HEp‐2 cells, an epithelial cell line derived from a human laryngeal carcinoma. HEp‐2 cells have replaced organ sections in IIF assays as they facilitate the observation and identification of many autoantibodies, primarily because these cells have larger nuclei and grow as a flat monolayer, and target antigens are expressed in various stages of the cell cycle [35, 134]. Moreover, HEp‐2 cells allow not only the detection of antibodies to nuclear antigens but also to cytoplasmic and mitotic components, which are equally clinically relevant; therefore, the term ANA, used in its literal sense, may be reductive [71, 133, 135, 136, 137, 138]**.**

#### 3.2.1 Performance of HEp‐2 IIF for ANA Detection

HEp‐2 IIF not only allows for the determination of the presence or absence of ANAs, but also provides other valuable information, such as their staining patterns and the antibody titer that provides a positive test result [135, 139].

**FIGURE 2:**
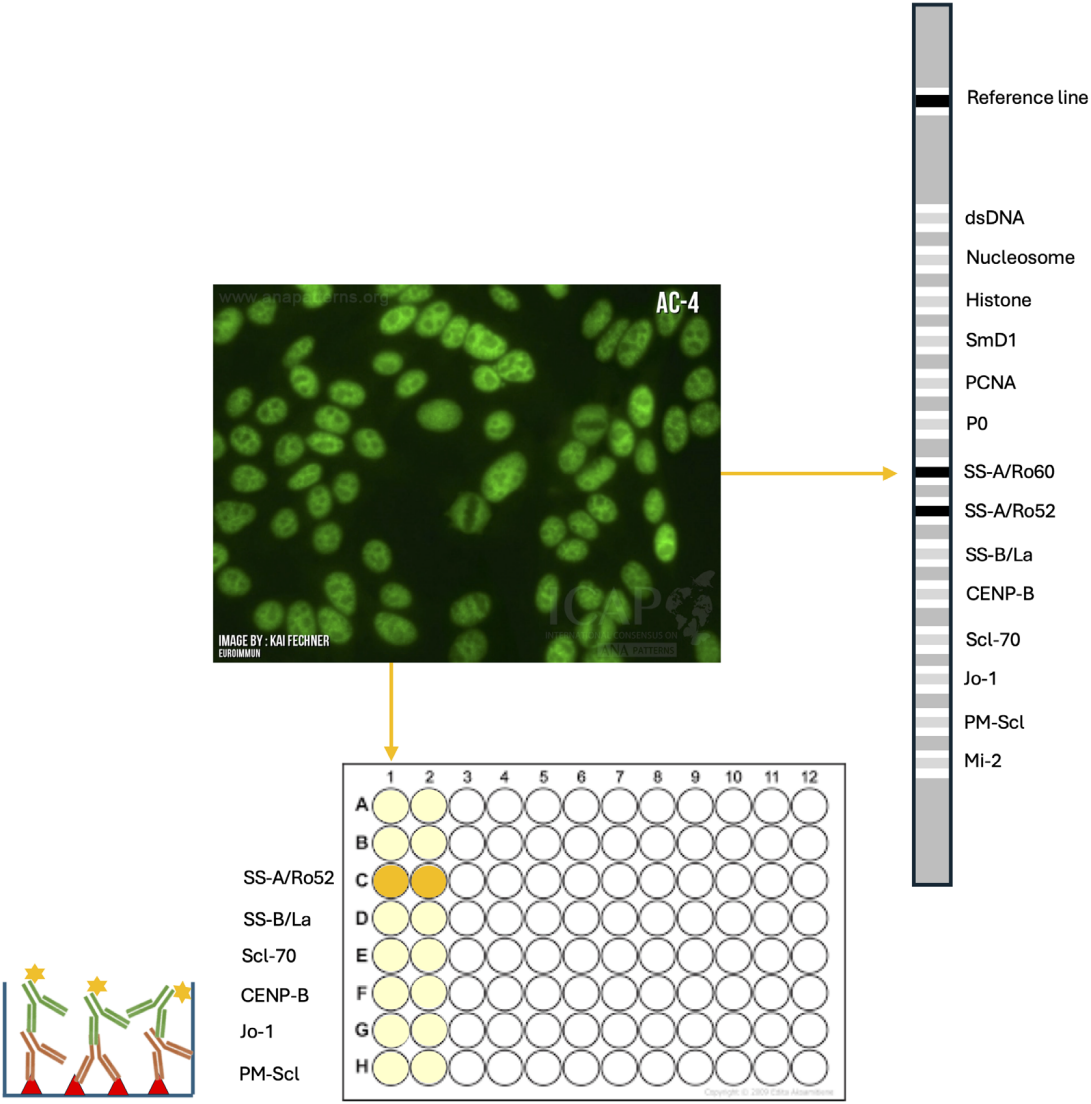
Indirect immunofluorescence (IIF) is the gold standard technique for ANA screening: the observed fluorescence pattern provides insight into ANA specificities. However, the identification of specific autoantibodies for a definitive diagnosis necessitates further characterization using antigen‐specific solid‐phase immunoassays (SPAs).

The primary ANA staining patterns are homogeneous, speckled, nucleolar, and centromere. Patterns can help determine the antibody specificities, but they are not sufficient, and further characterization using antigen‐specific solid phase immunoassays (SPAs) is recommended (Figure 2) [43, 140, 141, 142]. A first attempt at standardization of the nomenclature of the staining patterns has been made by the International Consensus on Antinuclear antibody Pattern (ICAP ‐ https://www.anapatterns.org/index.php), acknowledging that the patterns observed in the daily diagnostic workup are not always clear and easily attributed [136, 138].

The HEp‐2 IIF assay was defined as the gold standard for ANA screening by the American College of Rheumatology (ACR) because of its high sensitivity and intrinsic ability to detect a broad spectrum of autoantibodies compared to SPAs [143, 144, 145]. However, the assay suffers from low specificity, because ANAs are present also in nonrheumatic diseases and healthy individuals, and from low reproducibility across different manufacturers and laboratories. Several factors contribute to this variability, the first being the lack of harmonization between the many commercially available kits, particularly in the protocols employed to grow the HEp‐2 cells used, to prepare the slides, and the diverse nature of the reagents provided, parameters that influence the exposure of antigens and their epitopes in the cells. Inconsistent outcomes may impact diagnostic and clinical evaluations, inclusion in clinical trials, or the prescription of therapeutic agents [141, 146, 147]. IIF is also a time‐consuming technique, and the operator‐dependent interpretation of the results contributes to their variability [148]. More recently, advancements such as automated slide processors and computer‐aided diagnosis (CAD) have helped reduce subjectivity and improve reproducibility. However, some of these issues are still a matter of debate, as, on the one hand, the manual operator is not yet completely avoidable [149, 150]; on the other, we’re facing a near future where artificial intelligence and machine learning will increasingly be part of the analytic strategies in clinical medicine [151, 152].

#### 3.2.2 Perspectives in ANA Detection

The recent improvements in SPA and the limitations of HEp‐2 IIF for ANA screening have led many to reconsider its status as the gold standard: when it was appointed, SPAs were mainly based on the ELISA platform and had inferior performances, especially in sensitivity, but were spreading in clinical laboratories for their higher throughput and faster results [153]. However, ELISAs are now being replaced by more accurate chemiluminescent (CLIA) and fluoroimmunoenzymatic (FEIA) methods, which have proved to sometimes perform in a comparable or even superior way depending on the disease [71]. Moreover, the possibility to automate these technologies contributes to reducing sample processing time, thus increasing the productivity of the laboratories, and optimizing assay standardization [70]. Also, multiplexed assays such as ALBIA and PMAT have been developed and enable the simultaneous measurement of specific autoantibodies directed towards various autoantigens with a high degree of automation. Finally, the presence of commercial assays based on the line immunoblot technology, which allow the simultaneous detection of antibodies on a membrane strip (Figure 2) give an alternative strategy to identify disease‐associated antibody specificities [71, 154]. However, solid‐phase ANA assays are limited by their finite antigen panels, typically covering only around 15 major ones, which exclude rare specificities only detectable by IIF on HEp‐2 cells [154]. Moreover, the source of the antigen poses an additional challenge, as recombinant proteins may lack native PTMs or conformational epitopes that are recognized in IIF. Generally, despite their operational advantages, multiplex platforms introduce important analytical challenges, including complex calibration requirements, the need to optimize shared assay conditions for heterogeneous analytes, and increased susceptibility to cross‐reactivity and interanalyte interference, which may compromise sensitivity and specificity, particularly as panel size increases [65, 67, 69].

Overall, the diagnostic performances of these SPEs and their comparison with Hep‐2 IIF are still matters of debate. The consensus is that the combined use of IIF and SPAs for ANA screening enhances diagnostic accuracy and efficiency as they can provide complementary information: the likelihood ratio for AARDs is increased with double positivity (Figure 2) [71, 144, 150, 154, 155, 156]**.**

### 3.3 Autoantibodies in Autoimmune Hepatitis

In autoimmune liver diseases, an initial ANA screening is recommended by IIF on triple rodent tissue. However, a positive test should be followed up with HEp‐2‐IIF to characterize the fluorescence pattern, as ANAs are present in 75% of type 1 autoimmune hepatitis (AIH) patients [157], as well as 30%–50% of patients with PBC [158].

Antismooth muscle antibodies (SMA) were associated early with AIH and show three different staining patterns on kidney tissue: staining of arterial vessels (V), glomerular mesangium (G), or kidney tubules (T). While the V pattern is not disease‐specific, the VG and VGT are highly indicative of type 1 AIH, particularly when detected alongside ANA [157]. The autoantigens recognized by SMA are structures of the cytoskeleton: the major target is filamentous actin (F‐actin), a highly polymerized protein that is specific for AIH, as it is present in 80% of patients showing VGT‐pattern, with recognized epitopes being mainly conformational. The structural complexity of polymeric F‐actin and the loss of its native conformation during extraction may explain the variable performance of ELISA in detecting SMA. IIF continues to be the only method capable of reliably detecting the full spectrum of AIH‐1–related antigenic targets, although IIF techniques vary among laboratories and lack standardization. Efforts to establish nonoperator‐dependent assays, such as ELISA, employing other cytoskeletal proteins as antigens, have so far shown lower sensitivity than IIF for AIH‐1. Other antigenic targets include vimentin, tubulin, and desmin, although many still have to be characterized at the molecular level and thus are not considered in molecular tests based on purified or recombinant antigens [159, 160]. Although ANA and/or SMA, typically at low titers, may also be detected in patients with chronic hepatitis B or C, these antibodies generally lack specificity for F‐actin in such cases [161].

Antiliver kidney microsomal (LKM) antibodies are the serological marker of type 2 AIH. Of the three isoforms, in particular, anti‐LKM1 are found in up to 70% of AIH‐2 patients. The main antigenic target is the 2D6 isoform of the cytochrome P450 enzyme (CYP2D6), but also protein disulfide isomerase ERp57 and carboxylesterase 1 (CES1) are recognized [159, 162, 163]. Epitope mapping has identified several immunodominant regions, particularly amino acids 193–212, 257–269, and 321–351, recognized in most AIH‐2 patients.

Anti‐LKM1 are considered diagnostic for AIH‐2 in the absence of a hepatitis C virus (HCV) infection because some anti‐HCV antibodies cross‐react with a conformational epitope on CYP2D6 spanning the amino acids 254–288 containing the immunodominant epitope DPAQPPRD (263–270). This suggests that HCV infection might also play a role in the etiology of AIH through mechanisms like molecular mimicry [164, 165, 166].

Antiliver cytosol (LC1) antibodies, directed at formiminotransferase cyclodeaminase, a polymeric bifunctional enzyme involved in folate metabolism, are detected in about 30% of AIH‐2 patients, and in a third of cases, they are solely detected (the unique antibody specificity), so it is essential to include them in an initial screening [159].

#### 3.3.1 AIH‐Relevant Antibodies Detection

IIF allows the simultaneous detection of the major liver‐related autoantibodies, with the advantage of the possibility to detect autoantibodies whose target antigens are still unknown. While being the gold standard, the technique has several drawbacks. When performing IIF on triple rodent tissue, anti‐LKM1 stains brightly the hepatocyte cytoplasm and the proximal renal tubules, but not the stomach tissue. Anti‐LC1 antibodies also stain the hepatocyte cytoplasm, but not the cell layers around the central vein, and thus can be masked by anti‐LKM1 antibodies [167]. The use of only kidney tissue or misprepared substrates can lead to the wrong interpretation of results, confusing the staining.

Moreover, IIF is a time‐consuming, operator‐dependent, laborious technique, and it requires high‐quality substrates. Identifying the molecular targets of these antibodies has allowed the development of molecular‐based assays that use recombinant or purified antigens, particularly in the case of AMA, anti‐LKM1, and anti‐LC1, which are now commercially available and widely used. The common consensus is that SPAs should not be used as a screening tool, but to confirm IIF results [163, 168, 169]. For these reasons, other antigen‐specific techniques, such as immunoblot or counter‐immunoelectrophoresis, and solid‐phase assays based on purified or recombinant antigens have been established for their detection. In particular, antisoluble liver antigen antibody (anti‐SLA), the only AIH‐specific antibody, cannot be detected by standard IIF, but only through solid‐phase assays, most commonly competitive ELISA [161, 163, 170, 171].

As previously stated in paragraph 3.3., AIH is categorized into two main subtypes based on the serological profile. AIH type 1 (AIH‐1) is identified by the presence of SMAs and ANAs. Although SMAs, found in approximately 35% of AIH‐1 patients and 60% when combined with ANA, can also be detected in various other infectious, rheumatologic, or hepatologic disorders, the antibodies against their specific target, filamentous actin (F‐actin), are highly characteristic of AIH‐1. In contrast, AIH type 2 (AIH‐2) is defined by the detection of anti‐LKM type 1 (anti‐LKM1) and/or antiliver cytosol type 1 (anti‐LC1) autoantibodies [157, 159, 172]. While a gold standard for SMA F‐actin identification is still missing, international consensus identifies IIF on rodent tissue as the basic autoantibody test. On the other hand, reliable solid‐phase immunoassays (SPAs) that specifically target F‐actin, such as ELISA and immunoblotting, have been validated, with ELISA now included in updated diagnostic scoring. These SPAs offer distinct clinical advantages: ELISA provides quantitative data ideal for monitoring disease activity and treatment response and is suited for high‐throughput labs. On the other hand, immunoblotting's capacity for simultaneous detection of multiple autoantibodies makes it a valuable tool for confirming F‐actin specificity together with other antigens, improving both the accuracy and efficiency of AIH diagnosis in clinical practice [172, 173].

### 3.4 Primary Biliary Cholangitis

**FIGURE 3:**
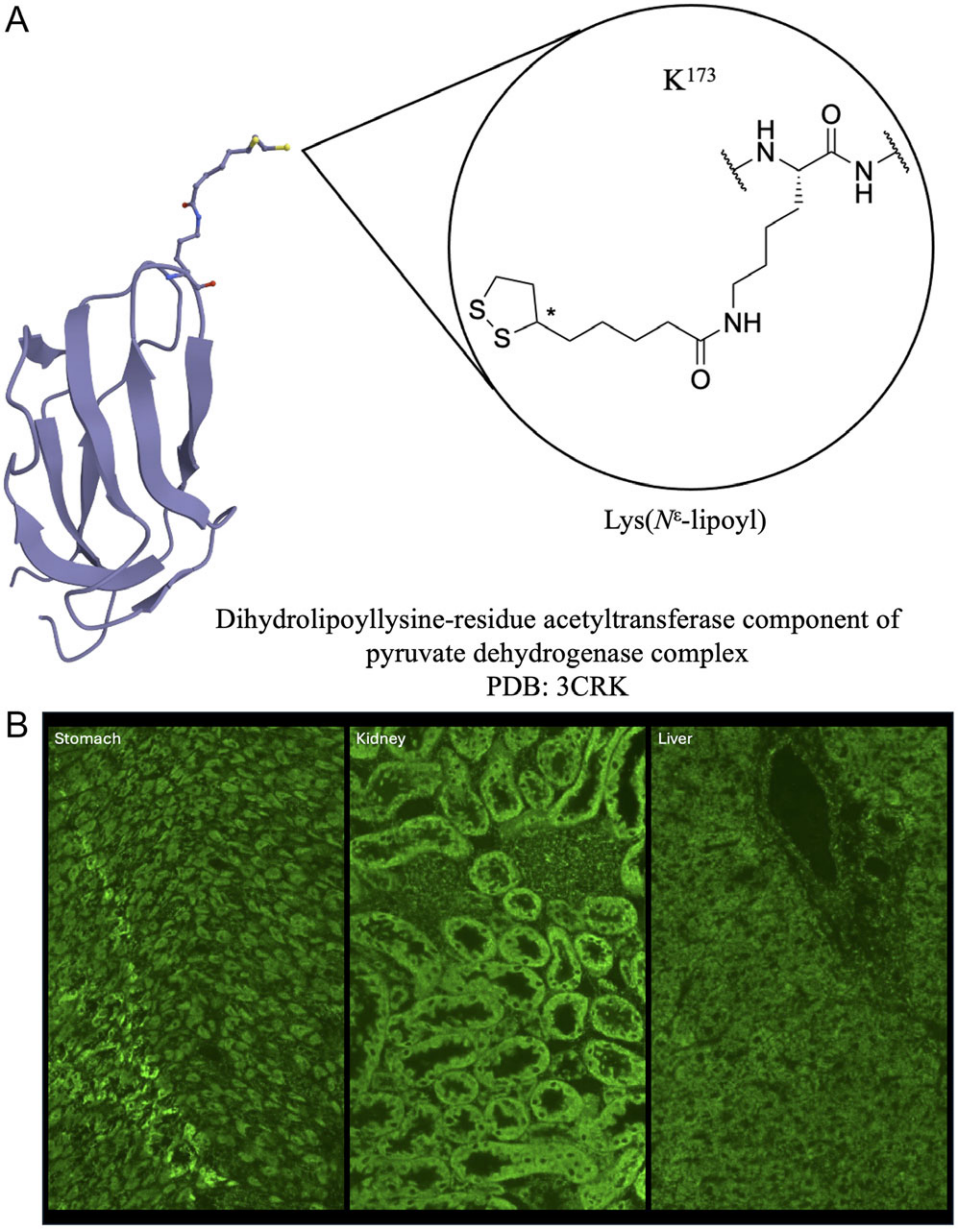
Antimitochondrial antibodies (AMAs) are highly specific for primary biliary cholangitis (PBC) as they are detected in 90%–95% of patients. (A) AMAs mitochondrial antigens located in the inner membrane, particularly the E2 subunits of the 2‐oxo acid dehydrogenase complexes (2‐OADC), with the pyruvate dehydrogenase complex (PDC‐E2) being the major AMA antigenic target. The immunodominant epitope of these antigens contains a motif bearing an essential post‐translational modification (PTM), lipoylation of lysine 173, emphasizing the critical role of the lipoyl domains for AMA recognition. (B) Immunofluorescence staining pattern of AMA shown on mouse tissue sections: stomach (left), kidney (middle), and liver (right).

AMAs are highly specific for PBC, and they are detected in 90%–95% of patients [174, 175]. In IIF on rodent liver, kidney, and stomach tissue, AMAs afford a characteristic fluorescence pattern with granular cytoplasmic staining of the renal tubular cells, gastric parietal cells, and hepatocytes (Figure 3B) [176].

These autoantibodies target specific antigens located in the inner mitochondrial membrane, in particular the E2 subunits of the 2‐oxo acid dehydrogenase complex (2‐OADC) [177, 178, 179]. The major antigenic target is the E2 subunit of pyruvate dehydrogenase (PDC‐E2), with other mitochondrial autoantigens being the E2 subunits of the branched‐chain 2‐oxo acid dehydrogenase complex (BCOADC‐E2) and oxoglutarate dehydrogenase complex (OGDC‐E2), as well as the E3 binding protein (E3BP). The immunodominant epitope is precisely mapped within the lipoic acid binding domain (lipoyl domain) of the E2 subunits, which contains an essential PTM, lipoylation of the **ε** group of lysine 173. The domain is exposed on the surface of the molecule, with the lipoic acid at the tip of the β‐turn structure [180, 181] (Figure 3A). The modification is both essential for the enzymatic activity of the proteins and crucial for the recognition and binding by AMAs [158, 182]. The antigenicity of the lipoyl domain may be partly explained by its distinct conformation, combined with the ability of lipoic acid to rotate via a “swinging arm” relative to the main PDC‐E2 molecule, as well as the dynamic opening and closing of the lipoic acid disulfide bond exploited in the enzymatic process during oxidative phosphorylation. However, this reactivity renders these antigens prone to aberrant modifications, thereby generating novel antigens that lead to loss of tolerance and consequent AMA recognition [180, 181]. Indeed, it was found that some xenobiotic‐modified lipoic derivatives of PDC‐E2 are more reactive to AMA than the native lipoyl PDC‐E2: in genetically predisposed individuals, prolonged exposure to electrophilic agents such as aspirin and acetaminophen could trigger or accelerate the loss of immune tolerance to PDC‐E2, ultimately resulting in PBC [183].

The recommended and most common method for AMA detection is IIF on cryostat sections of rat liver, kidney, and stomach as the substrate [177, 178].

Testing serum samples using other assays is necessary to confirm uncertain IIF results and to determine antibody specificities. Western immunoblotting (W‐IB) is one of the most used techniques due to its higher sensitivity and specificity compared to IIF [184]. The antigen sources for W‐IB are mitochondrial preparations from bovine or porcine heart, which are electrophoretically separated on sodium dodecyl sulfate‐polyacrylamide gels and transblotted onto nitrocellulose filters to show the major distinctive 74 kDa (PDH‐E2), 52 kDa (BCOADC‐E2) and 48 kDa (OGD‐E2) bands when incubated with PBC patients’ serum [176, 177].

However, W‐IB is a time‐consuming and qualitative technique, so automated and quantitative antigen‐specific assays, such as ELISA or PMAT, are taking its place [185, 186]. The latest ELISAs employ both purified PDC and a recombinant fusion protein (MIT3) consisting of the major immunodominant epitopes of the three main AMA targets [187]. This results in improved sensitivity compared to the single‐antigen ELISA or IIF since it is possible to detect both the most and less frequently recognized antigens [188].

### 3.5 Anticitrullinated Peptide/Protein Antibody ELISA for RA

The main antibodies involved in the diagnosis and classification of RA are the rheumatoid factor (RF) and the anticitrullinated protein/peptide antibodies (ACPA) [189]. The major role played by these antibodies in the diagnosis of RA is highlighted in the 2010 ACR/EULAR classification criteria, as detection of high levels of RF or ACPA accounts for up to 50% of the score required to diagnose RA [190].

RF was the first antibody linked to RA, and it is directed against the Fc portion of IgGs. The RF test is highly sensitive but not specific for RA, as RF can also be detected in other rheumatic, infectious, or chronic diseases, as well as in some healthy individuals, especially among the elderly. The detection of RF is commonly performed using methods such as nephelometry, turbidimetry, or ELISA, the latter having the advantage of differentiating between IgM, IgG, and IgA isotypes [191].

**FIGURE 4:**
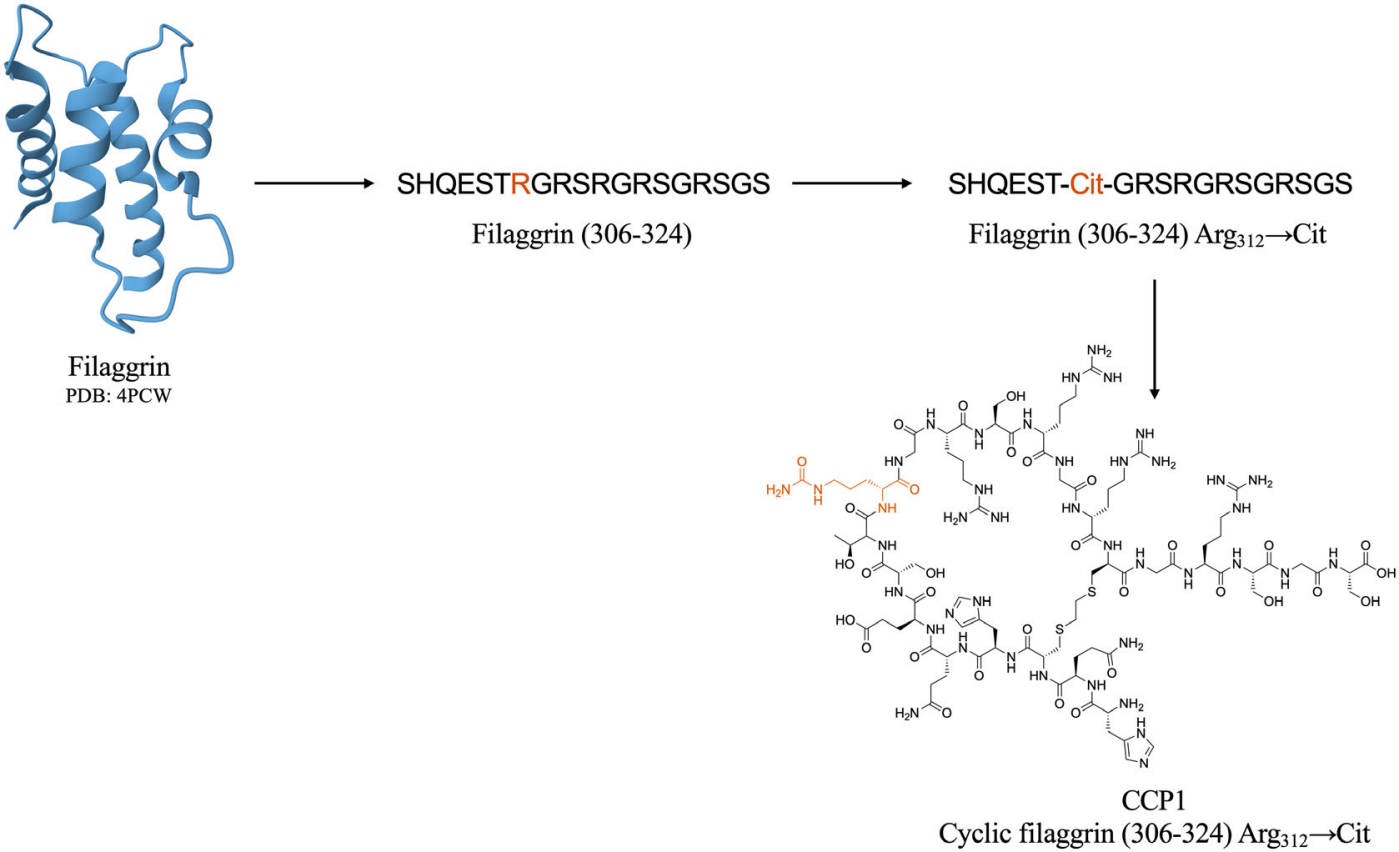
From filaggrin to the CCP1: Citrullination of the antigens is essential for recognition by RA‐specific autoantibodies. Citrullinated filaggrin was one of the first antigenic targets of anticitrullinated protein/peptide antibodies (ACPA) to be determined. Thus, citrullinated peptides based on its sequence were employed as antigenic probes. Cyclization of the peptide improved the assay's performance, leading to the development of the anti‐CCP1 test and the later generations.

ACPA are antibodies that recognize peptides and proteins containing citrulline residues, where citrullination is a PTM of an arginine residue to citrulline by enzymatic deimination [192]. ACPA were introduced in 1998 when Schellekens et al. demonstrated that citrulline is essential for the antigenic epitopes to be recognized by RA‐specific autoantibodies [193]. Their first ELISA based on citrulline recognition used synthetic citrullinated linear peptides from the sequence of human filaggrin, a previously identified antigenic target in RA, and showed high specificity but low sensitivity. To improve the performance, they substituted the linear peptide for a cyclic one, able to expose better the citrullinated epitope [194]. This first generation of anticyclic citrullinated peptide ELISA assay was named anti‐CCP1, and it was able to detect antibodies in 68% of RA sera with a specificity of 98% (Figure 4) [195]. However, CCP1 still used a filaggrin‐derived cyclic peptide as the antigenic substrate and filaggrin is not present in the synovial joint. Thus, about 12 million cyclic peptides from citrullinated synovial proteins were screened with RA sera, and the best ones were incorporated into a new assay, known as the second‐generation CCP test (CCP2 test), which showed improved performance compared to the CCP1 test and was useful in assessing the prognosis of the disease [196, 197]. It became commercially available in 2002 and it is still the most commonly used assay for the diagnosis and prognosis of RA [191, 195, 198].

Several new ACPA tests have become commercially available more recently. The third generation of anti‐CCP (anti‐CCP3) tests contains antigens with multiple citrullinated epitopes and has comparable performance to the anti‐CCP2 test in patients with early and established RA [199, 200]. A variant, the anti‐CCP3.1, was also developed, which can detect both IgA and IgG antibodies improving the sensitivity while maintaining similar specificity as anti‐CCP2 and anti‐CCP‐3 [195, 201].

Other synthetic citrullinated peptides are recognized by ACPA. VCP1 and VCP2 are multiple antigen peptides (MAPs) whose sequence is derived, respectively, from Epstein‐Barr nuclear antigen 1 and 2 (EBNA‐1/ EBNA‐2), which contain repeats of arginine residues, substituted with citrulline in the peptides, surrounded by neutral and small amino acids [202, 203]. Indeed, the Epstein‐Barr virus (EBV) is thought to have a role in the pathogenesis of RA [204, 205]. HCP1 and HCP2 are also MAPs, but their citrullinated sequence is derived from histone‐4 from activated neutrophils [206]. These four peptides are sensitive and specific antigens for the diagnosis of RA, and combining of HCPs and VCPs may increase the sensitivity in detecting both monospecific and cross‐reactive ACPAs [207].

Furthermore, both anti‐VCP and anti‐HCP antibodies are detectable in RA patients before symptom onset and predict disease development [208] and organ involvement [207].

The anti‐MCV test is based on mutated and citrullinated vimentin, a cytoskeletal protein and one of the first cellular proteins to undergo citrullination. However, the anti‐MCV is more sensitive, but less specific, and has lower diagnostic accuracy than anti‐CCP in RA [209].

Finally, the anti‐ENO CEP‐1 is a solid phase assay based on a citrullinated epitope of alpha enolase synthesized in the cyclic form may represent another way to detect ACPA with a specificity and a sensitivity of 83% and 65%, respectively [210].

Although ACPA assays were developed as ELISA, nowadays they are available for several automated analyzers based on CLIA, electrochemiluminescence (ECLIA), and FEIA [191, 211].

### 3.6 Antiphospholipid Syndrome

**FIGURE 5:**
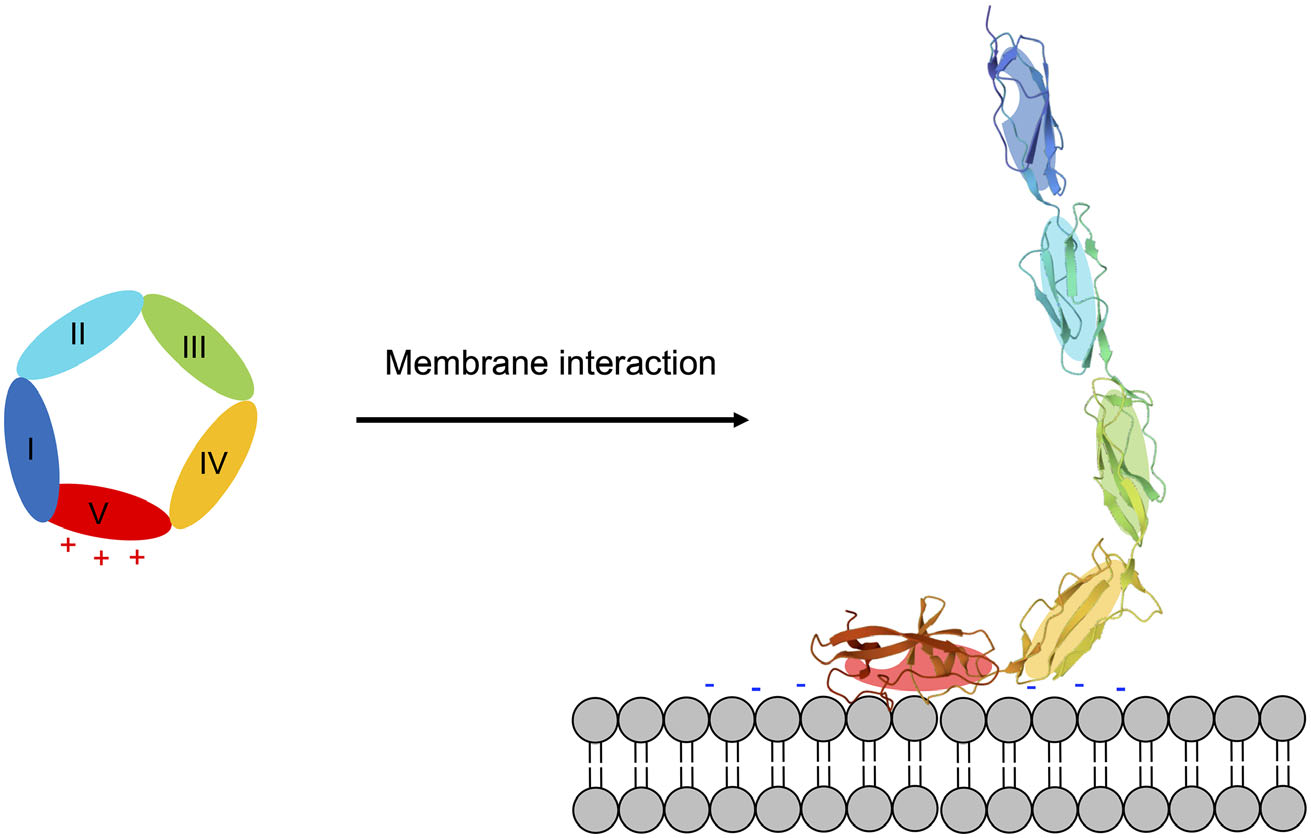
Structural transition of β2‐glycoprotein I: When the protein interacts with CL‐containing membranes, it undergoes a conformational change from a circular conformation, in which domains I and V interact, to an open conformation, revealing new antigenic sites and triggering the production of autoantibodies, a hallmark of APS.

APS is an autoimmune disorder characterized by the presence of antiphospholipid antibodies (aPL), autoantibodies directed against phospholipids, or, more specifically, phospholipid‐binding cofactor proteins, that lead to thrombosis in veins and arteries and pregnancy complications in patients [212]. aPL is a group of many autoantibodies directed against several different antigens [213]. In particular, the primary antigen in APS is β2‐glycoprotein I (**β**2GPI), a plasma glycoprotein made up of five repeating homologous domains (DI to DV), of which the fifth C‐terminal one (DV) contains a large lysine loop that allows the interaction with anionic phospholipids in cell membranes, like membrane‐associated cardiolipin (CL). Exposure to such surfaces induces a structural transition of **β**2GPI from a circular fold, in which domain DI and DV interact, to an open “fishhook” one in which the two domains are exposed, possibly displaying cryptic antigenic sites (Figure 5) [214].

Almost 20% (19%) of the molecular weight of **β**2GPI is composed of glycans, with four *N‐*glycosylation sites and one *O‐*glycosylation site. Glycans are essential in maintaining the looped conformation, while deglycosylation promotes the transition to the open conformation, favoring the interaction with anionic surfaces [215].

In APS patients, a decrease in sialylated triantennary glycans and an increase in sialylated biantennary glycan structures were observed, raising the possibility that these changes in glycosylation pattern alter stability and folding of **β**2GPI and thus may play a significant role in the generation of antibodies in APS [216, 217].

APS classification criteria include not only anti‐β2 glycoprotein I antibodies (aβ2GPI), but also lupus anticoagulant (LAC) and anticardiolipin antibodies (aCL) [218].

LAC defines a heterogeneous group of antibodies that inhibits phospholipid‐dependent coagulation reactions that, despite the name, are associated with an increased risk of both venous and arterial thrombosis and pregnancy loss, and the “anticoagulant” effect is exclusively observed during in‐vitro assays [219]. Since the LAC assay detects all antiphospholipid antibodies simultaneously, antigen‐specific antibodies can be detected with solid‐phase assays [220].

Anti‐cardiolipin antibodies and anti‐β2 glycoprotein 1 antibodies are directed respectively against cardiolipin, a phospholipid contained in cell membranes [221]. However, aCL ELISA tests are β2GPI‐dependent and thus actually detect anti‐β2GPI antibodies, as β2GPI in the reagents or the patient serum will bind to the cardiolipin (CL)‐coated plate. It is essential to avoid the detection of antibodies directly targeting CL, as they are usually due to infectious diseases [222, 223]. The aCL and anti‐β2GPI assays are commonly based on ELISA, as reflected in the 2023 ACR/EULAR APS classification criteria, which continue to rely on standardized ELISA measurements to evaluate antibody levels [218].

The anti‐β2GPI ELISA involves coating purified β_2_GPI directly onto an irradiated plate. Conceptually, this more selective assay should identify more clinically relevant antibodies than the aCL assay, but there is evidence suggesting that human β2GPI coated on a plastic surface may expose different epitopes, thus reducing the assay specificity [224]. Moreover, it is more limited, as it cannot detect antibodies directed at other phospholipid‐binding proteins that may also be clinically relevant [212, 225].

Newer techniques, such as chemiluminescence, fluoroimmunoenzymatic, and multiplex flow immunoassays, are substituting ELISA due to their harmonized protocols, higher automation, and greater analytical sensitivity, the latter leading to higher antibody titers compared to ELISA. This determines classification issues, considering that the ELISA thresholds used in classification criteria and automated platforms results are not correlated [222, 223, 226, 227, 228, 229]. However, the exclusion of non‐ELISA aPL antibody assays in the new 2023 ACR/EULAR classification criteria may potentially hinder the diagnosis or the inclusion of some APS patients in clinical trials [230].

Recently, immunoassays evaluating antibodies directed against the domain 1 of beta 2 glycoprotein (Anti‐Beta‐2 GPI D1), the N‐terminal region of the molecule, have been introduced in the routine, given their ability to identify antibodies more related to thrombosis. Under this view, anti‐**β**_2_GPI‐D1 antibodies might represent a promising tool when assessing thrombotic risk in patients with APS [231].

### 3.7 ANCA in ANCA‐Associated Vasculitis

Antineutrophil cytoplasmic antibodies (ANCA)‐associated vasculitis (AAV) is a group of autoimmune diseases characterized by inflammation of small to medium‐sized blood vessels, primarily affecting organs like the kidneys, lungs, and upper respiratory tract. This condition is marked by the presence of ANCA, mainly targeting myeloperoxidase (MPO‐ANCA) or proteinase 3 (PR3‐ANCA), proteins expressed in neutrophils, in particular in azurophil granules. MPO is a cationic dimeric enzyme in which the two monomers, linked with a disulfide bridge, consist of a heavy chain containing a modified iron protoporphyrin IX group and a glycosylated light chain. MPO plays a key role in the innate immune response by catalyzing the production of hypohalous acids from hydrogen peroxide and halide ions, which helps kill pathogens [232, 233]. PR3, a serine protease, is a highly folded protein with four disulfide bridges that carries out multiple roles in the immune system [234, 235].

AAV includes diseases such as granulomatosis with polyangiitis (GPA), microscopic polyangiitis (MPA), and eosinophilic granulomatous polyangiitis (EGPA), with PR3‐ANCA being most common in GPA, while MPO‐ANCA is most common in MPA and in approximately half of patients with EGPA [236]. The old consensus stated that IIF should be used as the initial screening method and that only positive samples should be tested by antigen‐specific immunoassays [237]. IIF for ANCA screening is performed on ethanol‐fixed neutrophils, which can present two major staining patterns: a cytoplasmic pattern is usually linked with PR3 reactivity and a diagnosis of GPA, while a perinuclear pattern is associated with reaction against MPO and MPA diagnosis. After positive IIF results, antibody specificities are determined with specific immunoassays [238, 239].

However, the 2017 revised international consensus on ANCA testing recommends antigen‐specific immunoassays as initial ANCA screening [240]. Indeed, in the last decades, solid‐phase assays have improved significantly, particularly ELISAs. The earliest PR3 and MPO assays were direct ELISAs, in which the antigen binds to the plate well through hydrophobic interactions, which can alter its conformation and potentially hinder adjacent epitopes, leading to inconsistent performances and low sensitivity of the assay. Second‐generation ELISAs exploit antibodies as capture molecules to bind the antigen: this reduces the blocking of the epitopes by the plastic plate and ensures that the conformation is preserved, but ANCA directed against the epitope bound by the capture antibody are not revealed. Thus, the sensitivity is increased but is still limited. This led to the development of third‐generation ELISAs, in which the antigen is anchored through a bridging small molecule, namely and adaptor peptide, that preserves both conformation and epitopes, determining a superior performance compared to direct and capture ELISA [241, 242, 243, 244].

Additionally, other types of solid‐phase assays are now commercially available and have performances comparable to ELISAs [240, 245]. A study showed that, compared with ELISA, the Dot Blot method demonstrated excellent performance for PR3 detection, with 100% sensitivity, 98% specificity, and 99% agreement, while for MPO detection it showed lower sensitivity (53%) but high specificity (97%), resulting in an overall agreement of 86% [246]. In another study, the Dot Blot assay showed strong concordance with standard ELISA and superior specificity compared to IIF, supporting its clinical utility as a rapid and reliable diagnostic tool for detecting anti‐PR3, anti‐MPO, and anti‐GBM antibodies, particularly in patients with a high suspicion of AAV [247]. Moreover, a multicentre European Vasculitis Study Group (EUVAS) evaluation compared different antigen‐specific immunoassays, namely ELISA, FEIA, multiplexed flow immunoassay and CLIA, and found comparable accuracies between them, ranging between 0.944 and 0.954, with AUCs derived from ROC curve analysis between 0.936 (95% CI 0.912–0.960) and 0.959 (95% CI 0.941–0.976) [245].

### 3.8 Anti‐GAD Antibodies in Diabetes

Glutamic acid decarboxylase (GAD) is the enzyme that mediates the transformation of glutamic acid into the neurotransmitter **γ**‐aminobutyric acid (GABA) and is found in both neurons and pancreatic **β** cells. There are two isoforms of GAD, GAD65 and GAD67, which have molecular weights of 65 and 67 kDa, the former being a major target of islet cell autoantibodies, biomarkers for autoimmune diabetes [248, 249], together with insulin, protein tyrosine phosphatase‐like protein IA2 and zinc transporter‐8 (ZnT8) [250].

In the case of type 1 diabetes (T1D), anti‐GAD antibodies (GADA) are the most prevalent autoantibodies in prediabetic relatives and new‐onset patients, present in 70%–80% of cases, with titers decreasing over time [249, 251]. Anti‐GAD65 antibodies are also present in various neurological disorders, generally with a much higher titer than the one observed in T1D, but they recognize a linear epitope versus a conformational epitope in T1D [252, 253, 254].

Liquid‐phase radiobinding assays (RBA) are considered the gold standard for GADA measurement and are still in wide use, due to their high sensitivity and specificity. Nonetheless, commercially available nonradioactive assays, such as bridge‐ELISAs, are spreading in many laboratories, and their performance is comparable, if not better than RBAs [255, 256]. In the GAD65 bridging ELISA by RSR [257], microplate wells are coated with highly purified, recombinant GAD65. First, the patient's serum is added to each cavity, allowing any anti‐GAD65 autoantibodies present to bind the solid‐phase immobilized antigen via one epitope. After this incubation, biotinylated GAD65 is introduced, which subsequently binds to another epitope of the patient's antibody. The patient's antibody thereby forms a “bridge” linking the solid‐phase GAD65 to the biotinylated one. The detection of this ternary complex is then achieved with streptavidin–peroxidase conjugates, which specifically bind to the biotin moiety of the antigen [258]. Individuals with multiple types of islet autoantibodies are at a significantly higher risk of developing T1D than those with single or no autoantibodies. Because serially checking for these various autoantibodies is a labor‐intensive and costly process, a novel tool that can simultaneously examine multiple islet autoantibodies is highly desirable for both diagnosing and evaluating the future risk of T1D [259]. Multiplexed ELISAs using three or more antigens, such as GAD65, IA‐2 and ZnT8, spotted in the same plate well, have shown superior performance compared to the single antibody test [260, 261].

In addition to T1D, islet cell autoantibodies, and in particular anti‐GAD65 antibodies, are also biomarkers of latent autoimmune diabetes in adults (LADA), where they represent the most common T1D‐related antibodies with about 91% prevalence. LADA is a slowly evolving immune‐mediated diabetes characterized by adult age of onset, positivity to islet cell autoantibodies and temporary insulin independence, thus with clinical features intermediate between T1D and T2D. For this reason, LADA is often misdiagnosed as T2D due to its similar gradual onset and initial insulin independence; thus the detection of anti‐GAD65 antibodies is crucial for correct diagnosis and treatment [258, 262, 263].

### 3.9 Antitissue Transglutaminase (tTG2) Antibodies in Celiac Disease

CD is a chronic, immune‐mediated enteropathy triggered by dietary gluten in genetically susceptible individuals, in which the antibodies target both gluten, namely, deamidated gliadin peptides (DGP), and an autoantigen, tissue transglutaminase 2 (TG2), and the presence of such antibodies is correlated with gluten dietary intake. In CD, there is T‐cell reactivity against gluten peptides that undergo deamidation, curiously by TG2 itself, whereby certain glutamine residues are converted into glutamic acid, generating negatively charged gluten peptides that bind with high affinity to HLA‐DQ2 or HLA‐DQ8 on antigen‐presenting cells, driving a CD4+ T‐cell response in the lamina propria. So TG2 has a dual role as the modifying enzyme and the principal autoantigen [264, 265].

TG2 is a calcium‐dependent ubiquitous enzyme, which has the main biological function of crosslinking glutamine and lysine residues of a protein, resulting in the formation of isopeptidyl bonds, in a process named transamidation [264, 266]. The presence of anti‐TG2 antibodies in serum is a hallmark of CD.

Initially, the diagnosis was based on serological testing for antibodies against unmodified gliadin, in combination with IIF testing for endomysial antibodies (EMA) on monkey esophagus or human umbilical cord. However, while being standardized, this technique is both time‐consuming and labor‐intensive, requiring skilled technicians for results interpretation, leading to a certain degree of operator variability. After the identification of TG2 as the main antigenic target of EMA, solid‐phase assays of TG2 have become the most widely used tests, from manual ELISAs to automated fluorescence or chemiluminescence‐based immunoassays [267, 268, 269].

According to the European Society for Paediatric Gastroenterology, Hepatology and Nutrition (ESPGHAN), the anti‐TG2 IgA assay is the primary test, often combined with anti‐TG2 IgG or anti‐DGP IgG in IgA‐deficient patients or young children. Anti‐EMA are frequently used to confirm positive anti‐TG2 results or to support nonbiopsy diagnosis in children [270, 271].

Based on the hypothesis that a covalently bound complex between TG2 and DGP represents an in vivo‐generated neo‐epitope in CD, antigens based on recombinant tTG cross‐linked to selected gliadin peptides have been created to improve the detection of IgA and IgG antibodies [272, 273].

By profiling tissue transglutaminase isoforms, namely tTG3 and tTG6, it has been possible to extend the clinical spectrum of gluten‐related autoimmunity beyond the intestine. In fact, tTG3 has not only been correlated with age at CD diagnosis [274], but is one of the principal autoantigens in dermatitis herpetiformis and therefore serves as a key marker of cutaneous manifestations in CD, with circulating anti‐tTG3 antibodies also detectable in some patients without overt skin lesions [275, 276]. Moreover, antibodies to tTG6 identified by a classical ELISA [277] have been correlated to gluten‐related neurological disorders, evidencing an extraintestinal autoimmune response that may occur independently of classical enteropathy [278].

### 3.10 Multiple Sclerosis Immunoassays

MS is the most common autoimmune disease of the central nervous system (CNS), characterized by inflammatory demyelination, neuronal injury, and neurodegeneration. It is one of the most common causes of nontraumatic disability among young adults [279]. Although traditionally thought to be a T cell‐mediated disease, the remarkable success of B‐cell‐depleting therapies in MS patients has highlighted the central role of B cells in the etiology and progression of MS [280, 281].

Diagnosis is made by evaluating a combination of signs and symptoms, radiographic findings (mainly using magnetic resonance imaging (MRI)), and laboratory findings, such as the presence of CSF–specific oligoclonal bands (OCBs). Indeed, according to the 2017 revisions of the McDonald Criteria, in patients with a typical clinically isolated syndrome and clinical or MRI evidence of dissemination in space, the presence of CSF‐specific OCBs is considered diagnostic of MS [282, 283].

OCBs are a hallmark of MS, as they are detected in up to 95% of patients, although they are not exclusive to MS. The presence of OCBs in CSF in the specific clinical context is still the most reliable parameter to confirm the diagnosis of MS [284].

OCBs are highly concentrated immunoglobulin species, mainly IgG, and indicate abnormal intrathecal immunoglobulin production associated with increased disease activity and worse prognosis [285]. Moreover, the identification of IgM OCBs in MS patients is linked to a more aggressive disease course [286].

As the name suggests, the detection of OCBs is carried out using isoelectric focusing (IEF) and then immunoblotting or immunofixation to reveal the IgG bands. IEF is an electrophoretic technique that separates proteins or peptides based on their isoelectric points (pI – the pH at which a protein carries no net electrical charges). In this process, a sample of CSF and a paired sample of serum are applied to an agarose gel under a pH gradient so that during electrophoresis, proteins migrate to the point in the gel where their pI matches the pH of the gel.

If immunoblotting is used, proteins are then blotted to a membrane of nitrocellulose, which is then probed with specific antibodies that detect immunoglobulins. Alternatively, in immunofixation, specific antisera are applied directly to the gel after IEF to precipitate the immunoglobulins, making them visible as discrete bands [287, 288].

Despite extensive research into potential viral and autoantigen targets, the exact antigenic specificity of OCBs is still unclear. Thus, in addition to imaging techniques, such as MRI, and OCBs detection, current research is focused on validating several molecular biomarkers found in CSF and blood‐based biomarkers for diagnosis and prognosis [285, 288].

Antibodies against several candidate autoantigens, including oligodendrocyte‐ and myelin‐derived proteins, have been identified in patients with MS, thus indicating incomplete B cell tolerance to these CNS antigens. However, these findings have not been conclusive in demonstrating an MS‐specific antibody response to any single target. For instance, antibodies against the intracellular antigen myelin basic protein (MBP), a key autoantigen in experimental models, are detected not only in patients with MS but also in individuals with other neurological disorders and in healthy controls [289].

Recently, molecular mimicry between the EBV transcription factor EBNA1, where EBV has been strongly associated with MS and is considered a prerequisite for developing the disease, and CNS antigens, including anoctamin 2 (ANO2), glial cellular adhesion molecule (GlialCAM), Alpha‐B crystallin (CRYAB), and MBP have been demonstrated [290, 291, 292, 293]. In most cases, a high degree of sequence homology was observed between EBNA1 and these CNS antigens. Notably, these homologous regions are clustered within a specific segment of EBNA1 located between the second glycine‐arginine repeat and the C‐terminal DNA‐binding domain, and increased antibody reactivity against this region has been strongly associated with MS [294]. Moreover, anti PLP antibodies correlate with increased disease severity in MS and can recognize native PLP on oligodendrocytes, supporting a direct pathogenic role in demyelination [295].

Analysis of the humoral response to post‐translationally modified components of myelin in serum patients revealed that the β‐d‐glucopyranosyl moiety (Glc) linked to an Asn residue (N‐Glc) is fundamental for antibody recognition in an MS patients’ population [296, 297, 298]. This led to the development of a peptide antigenic probe termed CSF114(Glc), which, due to the peculiar type I′ β‐turn structure, optimally exposes the Asn(Glc) epitope, emphasizing the importance of this PTM, in detecting MS‐specific antibodies in a subpopulation of MS, using both ELISA and SPR techniques [85, 299, 300]. The Asn(Glc) modification is virtually absent in humans but can be found in prokaryotes in cell‐surface proteins such as adhesins, supporting the hypothesis of the involvement of an infection by an exogenous pathogen in MS pathophysiology. Indeed, it was found that a hyperglucosylated protein domain from adhesin protein of nontypeable *Haemophilus influenzae* (NTHi) termed HMW1, is preferentially recognized by antibodies from sera of an MS patient subpopulation [301, 302].

### 3.11 CBAs in Demyelinating CNS Syndromes

Antibody‐mediated disorders of the CNS are immune‐related neurologic conditions marked by the presence of neural autoantibodies targeting specific antigens expressed in the CNS and share various clinical and MRI features. In particular, autoantibodies directed at surface antigens are thought to be pathogenic as they are easily accessible to circulating antibodies [303, 304]. Aquaporin‐4‐IgG positive neuromyelitis optica spectrum disorder (AQP4 + NMOSD) and myelin‐oligodendrocytes glycoprotein antibody‐associated disease (MOGAD) have been recently identified as antibody‐mediated demyelinating autoimmune disorders of the CNS, distinct from MS, of which they were long considered subtypes, due to the clinical and radiological overlaps. However, a specific diagnosis is essential as these diseases have clinical features, treatment considerations, and prognoses different from MS. Biomarkers of these diseases are, respectively, autoantibodies targeting the aquaporin‐4 (AQP4) water channel and myelin oligodendrocyte glycoprotein (MOG) [305]. The membrane location of these proteins makes the correspondent antibodies excellent candidates to be detected via CBAs. Indeed, CBAs are the recommended method for testing both antibodies, according to international consensus [306, 307].

In 2005, AQP4 was proved to be the antigenic target of specific autoantibodies found in patients with NMOSD [308].

AQP4 is one of the 13 mammalian aquaporins, and it exists in two major isoforms, the full‐length M1 isoform and the shorter M23 isoform, differing from 22 N‐terminal residues. Like all AQPs, AQP4 forms tetramers that can be both homotetramers (either M1 or M23) and hetero‐tetramers (both M1 and M23) in membranes, with each monomer containing a water‐selective pore. The M1 isoform is mainly found as an individual tetramer, while M23 assembles in large supramolecular aggregates containing up to 600 M23‐AQP4 tetramers called orthogonal arrays of particles (OAPs) [309, 310].

Initial detection methods for anti‐AQP4 antibodies based on tissue IIF (TIIF) were later replaced by immunoprecipitation assays and ELISA, the latter offering improved sensitivity but also presented occasional false positives, indicating the need for more specific assays.

CBAs using HEK293 transfected with a plasmid encoding human AQP4 have the highest sensitivity and specificity. Although initially limited to specialized centers, the advent of CBA commercial kits allowed for widespread use of the assay [306, 311, 312, 313].

Several factors may potentially influence AQP4‐IgG CBA performance. It has been demonstrated that anti‐AQP4 IgG have a higher affinity for M23 than M1 as a consequence of OAPs formation by M23 AQP4 isoforms [314]. However, the best isoform to employ as antigen in an immunoassay is still debated, as M23 determines higher sensitivity, but also a higher signal‐to‐noise ratio [313, 315]. Overall, there may be slight variations in the performance of different AQP4‐IgG CBAs due to using the different M1 or M23 isoforms, or live instead of fixed cells, but the diagnostic accuracy of CBAs is still higher compared to other testing methods, also with strong agreement irrespective of antibody titer in live CBAs [305, 312].

However, ELISA is still the most easily accessible assay in many cases and offers the advantage over CBAs of being faster, easier to use, and can be automated. If the result is negative in patients with high clinical suspicion or for low and moderate positive results, further evaluation using CBAs should be considered. A new automated commercial ELISA that uses the M23 isoform has shown promising performance, comparable to CBAs [316, 317].

MOG is a transmembrane glycoprotein that is uniquely expressed on the surface of the myelin sheath, specifically on the membrane of oligodendrocytes in the CNS. Still, it is a quantitatively minor component of myelin (0.05%) and its biological role is still unknown [318, 319]. The N‐terminal region of MOG forms an Ig‐V fold consisting of two antiparallel β‐sheets. It also presents one N‐linked glycosylation site on asparagine 31 [320, 321].

In early studies, when tested using ELISA or immunoblotting, anti‐MOG IgGs were observed with a higher incidence in MS patients. However, these techniques offered inconsistent and not reproducible results, and also considering the relatively high positivity rate among healthy controls, the detection of antibodies has been attributed to the recognition of non‐native MOG epitopes [50]. The development of CBAs for MOG IgG has been instrumental in identifying MOGAD as a distinct disease.

A study [322] suggests that the need for CBAs could be due to the bivalent binding of anti‐MOG antibodies, as the intracellular part of MOG included in the full‐length protein used in CBAs induces a clustering in which the spacing of the extracellular part of MOG allows bivalent binding. However, the manner in which the structure, quantity, or density of MOG molecules in vivo on oligodendrocytes facilitates MOG IgG binding in MOGAD remains unclear [323]. Nonetheless, it is thought that MOG can form dimers, and MOG tetramers where found to better recognize antibodies in a RIA [321, 324].

According to international guidelines, the assay should be performed using live cells expressing full‐length MOG (α1 isoform) and using either an IgG Fc or IgG1‐specific secondary antibody for detection to achieve the best assay performance [307]. However, in contrast with AQP4‐IgG, testing for MOG IgG with live CBAs seems to provide some advantage over fixed CBAs. In an international multicenter examination, a high degree of agreement across assays was achieved with clear positive and negative results, with slightly poorer results when a fixed CBA was included, demonstrating that some important conformational epitopes are masked by fixation of cells. The agreement on borderline positives, however, was poor and caution is needed with low‐positive MOG‐IgG as it can be found in 1% to 2% of controls [305, 325].

### 3.12 Myasthenia Gravis

Myasthenia gravis (MG) is an autoimmune disorder of the neuromuscular junction (NMJ) characterized by weakness and fatigue of the muscles. Disease‐specific autoantibodies target proteins at the NMJ. The major antigen is the nicotinic acetylcholine receptor (nAChR) of the muscle postsynaptic membrane. nAChR is a ligand‐gated ion channel that consists of five subunits, each comprising a large extracellular domain containing the ligand recognition site, and four helical transmembrane domains. Autoantibodies bind to epitopes in the extracellular domains of the nAChR subunits, especially a segment of the **α**1‐subunit, known as the main immunogenic region (MIR). The MIR is a loop made by amino acids 66–76, which may play a role in the pathogenicity of the antibodies targeting it as it allows bivalent binding, resulting in the crosslinking of the nAChR molecules [326, 327, 328].

Some other patients have instead autoantibodies targeting muscle‐specific kinase (MuSK), a postsynaptic molecule required for inducing AChR clustering and maintenance of the NMJ, or, in other cases, lipoprotein‐related protein 4 (LRP4), also involved in AChR clustering. These autoantibodies are considered disease biomarkers and are crucial in classifying patients into distinct subgroups [329, 330].

Radioimmunoprecipitation assay (RIPA) is the gold standard for AChR and MuSK antibody detection: its diagnostic sensitivity depends on MG subtype, while the specificity is nearly 100% in all cases. ELISA has been proposed as an alternative platform with the aim of eliminating the use of radioactive reagents, but it appears to have poorer diagnostic performance with possible false‐positive results. Live CBAs showed higher sensitivity and comparable specificity to RIPA, but they are limited to specialized centers. However, fixed CBAs for detection of MG‐specific autoantibodies have recently become available and are more easily implementable than live CBAs for routine diagnosis [331, 332]. The improved sensitivity of AChR CBAs is due to cotransfection of the cells with rapsyn, which induces the clustering of AChR molecules on the membranes of cultured cells, thus enabling the detection of low‐affinity antibodies also in patients with antibodies not detectable with radioimmunoassay. In the case of MUSK, better diagnostic performance are due to correct glycosylation and folding of the protein, and can be improved using IgG Fc secondary antibodies [331]. Few large, prospective studies comparing CBAs to RPA and ELISA for autoantibody detection in MG have been performed, but there is agreement that the advent of CBA marks a diagnostic breakthrough in MG [331, 332, 333].

## 4 Conclusions and Future Perspectives

Autoantibodies represent key molecular signatures in autoimmune diseases, providing insights into all aspects of the disease. From a clinical standpoint, autoantibodies play a crucial role in the diagnosis, prognosis, and therapeutic management of autoimmune diseases. Indeed, autoantibody profiling is essential for classifying diseases with overlapping clinical presentations. Moreover, certain autoantibody patterns correlate with disease severity or activity, and longitudinal monitoring of antibody titers provides a noninvasive measure of treatment response and risk of relapse [17, 18, 19, 20, 334]. The continual discovery of novel autoantigens and autoantibodies has deepened our insight into disease pathogenesis, unveiling molecular signatures that are unique to individual patients or subgroups. Considering the heterogeneity of manifestations between individuals in AIDs, patient‐specific antibody profiles are fundamental to enable early and precise diagnosis, improved prediction of disease course, and better targeted therapeutic interventions, supporting the principles of personalized medicine (PM). Technological progress has been instrumental in expanding the diagnostic and research potential of autoantibody testing. The selection of assay is largely determined by the nature of the target antigen. Autoantibodies recognizing intracellular antigens or linear epitopes are typically amenable to solid‐phase assays such as ELISA, CLIA, or line immunoassays because these antigens preserve sufficient structural integrity and epitope accessibility after immobilization on solid supports. On the other hand, autoantibodies directed against membrane proteins or conformational epitopes often lose their native structure outside their own environmental cellular context requiring specific techniques such as immunofluorescence (IF) or CBAs, which preserve 3D folding and post‐translational modifications. In this context, conventional methods, including IIF, ELISA, and immunoblotting, remain essential in clinical laboratories owing to their specificity and established validation. However, their low throughput and limited multiplexing capacity restrict their use in large‐scale serological profiling. The introduction of proteomics‐based multiplexed immunoassays, such as antigen microarrays and display technologies, has revolutionized the field by allowing the simultaneous detection of hundreds of autoantibody specificities within a single sample [19, 63, 335, 336].

Planar arrays maximize multiplexing capacity, often spotting thousands of unique antigens on glass slides, making them the preferred platform for initial discovery of novel autoantigens. Conversely, bead‐based arrays, which immobilize antigens on color‐coded microspheres that are read via flow cytometry, offer superior sample throughput, making them ideal for biomarker verification and validation. Antigen‐wise, we define two major types: protein arrays and peptide arrays. Protein arrays display full‐length proteins, often requiring production through cellular expression systems or cell‐free systems (like Nucleic Acid Programmable Protein Array), and are essential for detecting autoantibodies targeting conformational or discontinuous epitopes. Peptide arrays utilize shorter linear fragments (often 12–20 amino acids), are highly suited for fine‐mapping linear epitopes and easily incorporating PTMs (e.g., citrullination). Finally, next‐generation display technologies like Phage Immunoprecipitation Sequencing (PhIP‐Seq), displaying peptides on bacteriophage, and Rapid Extracellular Antigen Profiling (REAP), displaying proteins on yeast, utilize next‐generation sequencing to quickly profile autoantibody responses across potentially the entire proteome [336, 337, 338, 339, 340, 341].

In conclusion, the field of autoantibody detection is rapidly transitioning from simpler single‐parameter assays toward multiplexed and higher‐throughput platforms. These advances are not merely technical, but they represent a fundamental shift in how autoimmune diseases are diagnosed and managed. Understanding antibody specificities at molecular resolution, considering structural conformation, PTMs, and epitope diversity, will continue to refine PM, enabling clinicians to tailor management strategies to the unique immunological signature of each patient. The ongoing discovery of novel autoantigens and the refinement of multiplex immunoassays will continue to redefine the diagnostic landscape, translating serological complexity into actionable clinical insight.

## Funding

This work was supported by PhD fellowship to S. B., funded by the European Union ‐ Next Generation EU, PNRR MUR M4 C2 Inv. 1.5 of the National Recovery and Resilience Plan (PNRR), project ECS00000017 Tuscany‐Health Ecosystem‐Spoke 6 (CUP B63C2200068007), and Tuscany‐Health Ecosystem (Grant ECS_00000017).

## Conflicts of Interest

The authors declare no conflicts of interest.

## Data Availability

Data sharing not applicable to this article as no datasets were generated or analyzed during the current study.
